# Beta-Blocker and Calcium Channel Blocker Toxicity With BRASH Syndrome: A Case Report

**DOI:** 10.7759/cureus.33544

**Published:** 2023-01-09

**Authors:** Adolfo Martinez, Niket Shah, Andrew Kim, Kevin Watat, Sandeep Banga

**Affiliations:** 1 Internal Medicine, Michigan State University, East Lansing, USA; 2 Medicine, Sardar Vallabhbhai Patel (SVP) Hospital, Ahmedabad, IND; 3 Cardiology, Michigan State University, East Lansing, USA

**Keywords:** calcium channel blocker, beta blocker, case report, hyperkalemia, renal failure, bradycardia, brash syndrome

## Abstract

Atrioventricular (AV) nodal blockers have a wide variety of medical uses, including the management of hypertension and cardiac arrhythmias. Like any other drug, they can carry side effects and toxicity. We present a case of a patient with a constellation of findings consistent with bradycardia, renal failure, AV nodal blockade, shock, and hyperkalemia (BRASH) syndrome. A 75-year-old female with a history of paroxysmal atrial fibrillation and heart failure with preserved ejection fraction presented to the hospital with shortness of breath. She was discharged two weeks prior to the presentation from another hospital after being treated for atrial fibrillation with a rapid ventricular response. She was discharged on metoprolol and diltiazem. Upon presentation to the hospital, the patient was noted to be bradycardic and hypotensive with blood work notable for acute kidney injury and hyperkalemia, consistent with BRASH syndrome. She received a dose of intravenous (IV) glucagon followed by infusion and received epinephrine infusion. Once clinically stable, she was discharged with her home dose of metoprolol and a reduced dose of diltiazem with a close follow-up with cardiology. Early recognition of BRASH syndrome as a unique clinical entity rather than different pathologic conditions is important to improve morbidity and mortality in these patients.

## Introduction

Beta-blockers and calcium channel blockers (CCBs) can cause fatal complications in susceptible patients such as those with underlying kidney dysfunction. Bradycardia, renal failure, atrioventricular (AV) nodal blockade, shock, and hyperkalemia (BRASH) syndrome has been used to describe a constellation of clinical findings that may be induced by these AV nodal blocking agents [1]. The term BRASH syndrome is still not widely used, and there are still many knowledge gaps about this condition. Here, we present a case of a patient who developed BRASH syndrome while on diltiazem and metoprolol for her atrial fibrillation.

## Case presentation

The patient is a 75-year-old female with a past medical history of paroxysmal atrial fibrillation, chronic obstructive pulmonary disease, heart failure with preserved ejection fraction, stroke, and squamous cell carcinoma of the left lung who presented to the hospital with shortness of breath.

Two weeks prior to presentation, the patient was admitted to an outside hospital for heart failure exacerbation and new-onset atrial fibrillation with the rapid ventricular rate (RVR). She was started on diltiazem and metoprolol on discharge due to difficult rate control. There were no other medication changes that could predispose to acute kidney injury (AKI) or hyperkalemia.

The patient related that she began developing generalized weakness with increased shortness of breath one week prior to the presentation. At the hospital, she was found to be bradycardic (heart rate of 38), hypotensive, and hypoxic with symptoms of dizziness, lightheadedness, and syncope.

On the initial evaluation, the patient was found to have elevated aspartate aminotransferase (AST) and alanine transaminase (ALT), an acute kidney injury (AKI) (baseline creatinine was 0.8-1.0), and hyperkalemia (Table 1).

**Table 1:** Laboratory analysis results on admission to the hospital

Component	Obtained value	Reference Range and Units
Calcium	7.76	8.00-10.50 mg/dL
Alkaline Phosphatase	193	55-142 U/L
Bilirubin, Total	0.4	0.2-1.2 mg/dL
Protein, Total	5.7	6.0-8.0 g/dL
Albumin	3.4	3.6-5.0 g/dL
Blood Urea Nitrogen	42	6-23 mg/dL
Creatinine, Serum	1.47	0.60-1.40 mg/dL
Glucose	201	65-99 mg/dL
AST	468	10-40 U/L
ALT	325	3-45 U/L
Sodium	139	135-145 meq/L
Potassium	5.5	3.5-4.9 meq/L
Chloride	106	96/110 meq/L
CO2, Total	22	20-32 mmol/L
Anion Gap	11	2-16

**Figure 1:**
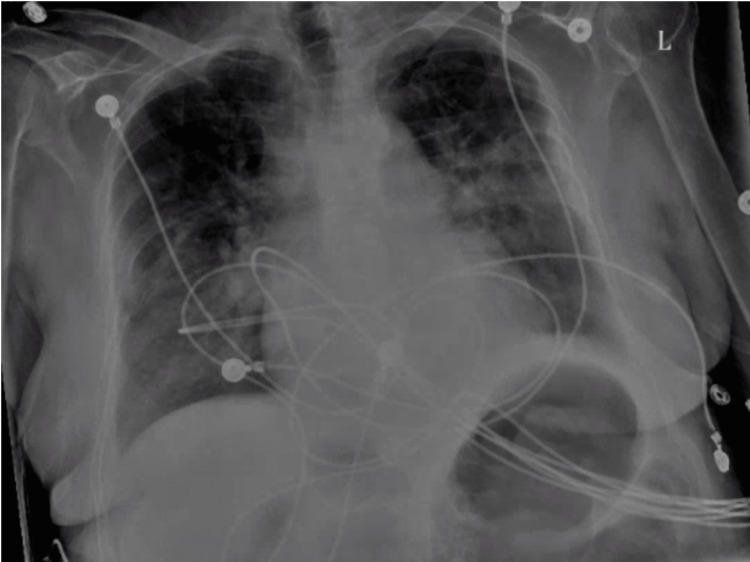
Chest X-ray on admission showing cardiomegaly with bilateral pulmonary edema Imaging obtained from the patient's chart

An initial chest X-ray showed bilateral lung opacities consistent with pulmonary edema (Figure 1).

**Figure 2:**
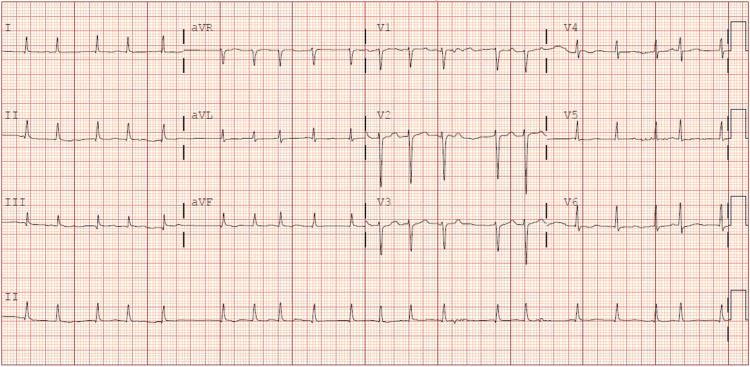
Electrocardiogram demonstrating atrial fibrillation with rapid ventricular rate Electrocardiogram obtained from the patient's chart

Diltiazem and metoprolol were immediately stopped. She received one dose of calcium gluconate and insulin with dextrose for hyperkalemia. Intravenous (IV) atropine for bradycardia was given with no improvement in her heart rate for which an epinephrine drip was started. She was also given a glucagon IV bolus followed by an infusion to counteract beta-blocker toxicity. Her acute kidney injury and hyperkalemia resolved in 24 hours after the discontinuation of the AV nodal blockers and with the epinephrine infusion. At the same time, she was also started on antibiotics for suspicion of pneumonia. She was admitted to the intensive care unit for close monitoring and patches were placed for potential temporary pacing if needed. The following day the patient went into atrial fibrillation with RVR (Figure 2), and this was consulted with an electrophysiologist.

At this point, the epinephrine and glucagon infusions were stopped and the patient’s metoprolol was resumed in a stepwise manner. However, she became hypotensive while still on atrial fibrillation with RVR, and digoxin was given for 1 day with a good increase in her blood pressure. After the patient was out of this critical status, she was also started on IV diuresis given the associated heart failure exacerbation. The patient was eventually discharged on her home dose of metoprolol and a reduced dose of diltiazem with a close follow-up with the cardiology service.

## Discussion

BRASH syndrome was first described in 2016 as a complex clinical entity that involves bradycardia, renal failure, AV node blocking, shock, and hyperkalemia. This constellation of clinical findings is seen in predisposed patients that are taking either CCBs or beta-blockers [1]. However, atypical variants have been also described with the use of other medications like ranolazine and even with coronavirus disease 2019 (COVID-19) infection [2,3].

It is important to note that the syndrome often goes unrecognized in patients that get admitted to the hospital (given that it’s a relatively new clinical term), which can delay prompt diagnosis and care [4]. Suspicion should be high in any patient with severe bradycardia and associated hyperkalemia [5]. Only a few case reports have been published in the literature. However, it seems that this condition is more common in elderly patients with underlying cardiac and kidney disease which have a predisposition for AV node agent side effects [6].

The pathophysiologic mechanism usually involves a certain degree of generalized hypoperfusion, which could be secondary to dehydration or sepsis [7]. Hypoperfusion can lead to renal failure, which can cause hyperkalemia and hemodynamic compromise [8]. In this given scenario, AV nodal agents can accumulate causing bradycardia, which can lead to a decreased cardiac output and reduced renal perfusion, which worsens kidney injury and hyperkalemia. However, it is important to know that beta-blockers are known to also cause hyperkalemia by reducing the activity of the NA+/K+ pump in the cellular membrane and disrupting sympathetic input on the release of renin [9]. The mechanism of how CCBs can induce and perpetuate hyperkalemia is less clear. The most commonly associated CCB is verapamil, which is known to induce hyperkalemia via the trans-cellular shift mechanism [10].

These mechanisms establish a vicious cycle in which renal failure increases the levels in the blood of these AV nodal medications and perpetuates hyperkalemia and bradycardia, leading to circulatory shock [5]. Given this complex pathophysiology and the multiple mechanisms involved, patients can have a very non-specific clinical presentation, including generalized weakness, shortness of breath, dizziness, or syncope [11].

**Figure 3:**
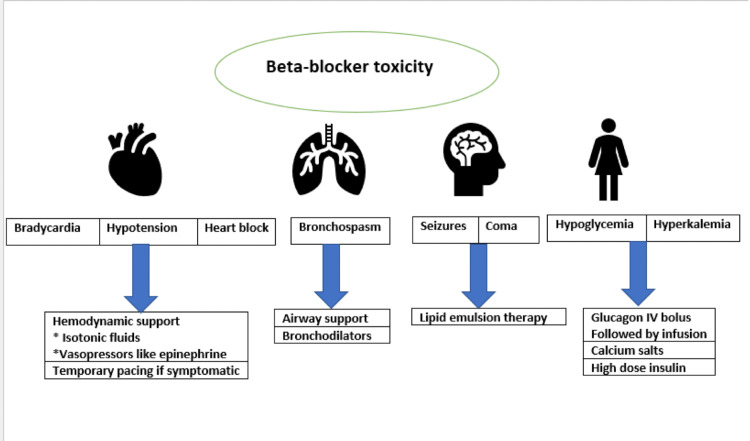
Presentation and management of beta-blocker toxicity Author's own creation

It is important to recognize all the components of the syndrome as a whole and not as isolated medical problems that need to be addressed. Therefore, clinicians should have a high clinical suspicion for correlating all these findings [12]. There is no established treatment for BRASH syndrome, but the first recommended step is to stop the AV nodal blocking agents that may continue the cycle mentioned above [13]. Upon stopping these medications, supportive measures need to be started (Figure 3).

For hyperkalemia, it’s important to start calcium gluconate (if it's severe and/or electrocardiogram changes are present) and other potassium-lowering measures. For bradycardia and shock, epinephrine and glucagon (if they are beta-blocker induced) can be considered. Glucagon reverses the beta blocker effect by activating adenylate cyclase, which causes an increase in cyclic adenosine monophosphate (cAMP) with a subsequent increase in intracellular calcium with enhanced depolarization and contractility [14]. If there’s no response to these measures, epinephrine can be considered, and it’s important to keep a low threshold for transcutaneous pacing [15]. It is important to know that atropine is not effective in BRASH syndrome because in this case, the bradycardia is not mediated by the vagus nerve [6]. Our patient had a very mild to no response with atropine and was eventually started on glucagon and epinephrine drip for one day after which her hemodynamic status significantly improved.

There is a gray area in the current medical literature when talking about the best time to resume AV nodal blockers in these patients. A benefits and risks discussion with patients who need these medications to improve survival (like our patient with a diagnosis of congestive heart failure) needs to be done. With our patient, metoprolol was resumed the next day after the patient was off the epinephrine drip and diltiazem was resumed afterward but in a smaller dose. These were resumed given her atrial fibrillation, which has been difficult to control in the past. On discharge follow-up, she has not had any recurrent symptoms.

## Conclusions

The early recognition and correlation of the clinical findings in BRASH syndrome are important given the devastating consequences that these patients can have. Once identified, it’s important to discontinue the AV node blockers and give supportive measurements for renal failure and hyperkalemia. Early recognition of this condition can help improve clinical outcomes in these patients.
